# Dysregulation of fibulin-5 and matrix metalloproteases in epithelial ovarian cancer

**DOI:** 10.18632/oncotarget.24484

**Published:** 2018-02-14

**Authors:** Dustin B. Manders, Hari Annavarapu Kishore, Adi F. Gazdar, Patrick W. Keller, Jun Tsunezumi, Hiromi Yanagisawa, Jayanthi Lea, Ruth Ann Word

**Affiliations:** ^1^ Department of Obstetrics and Gynecology, Green Center for Reproductive Biology, University of Texas Southwestern Medical Center, Dallas, Texas, USA; ^2^ Division of Gynecologic Oncology, Department of Obstetrics and Gynecology, University of Texas Southwestern Medical Center, Dallas, Texas, USA; ^3^ Hamon Center for Therapeutic Oncology, University of Texas Southwestern Medical Center, Dallas, Texas, USA; ^4^ Department of Molecular Biology, University of Texas Southwestern Medical Center, Dallas, Texas, USA; ^5^ Current address: Life Science Center, Tsukuba Advanced Research Alliance, University of Tsukuba, Tsukuba, Ibaraki, Japan

**Keywords:** extracellular matrix, tumor macrophages, MMP-7, cell adhesion

## Abstract

Fibulin 5 (FBLN5) is an extracellular matrix glycoprotein that suppresses matrix metalloprotease 9 (MMP-9), angiogenesis and epithelial cell motility. Here, we investigated the regulation and function of *FBLN5* in epithelial ovarian cancer (EOC). FBLN5 mRNA was down-regulated 5-fold in EOC relative to benign ovary. Not surprisingly, *MMP9* mRNA and enzyme activity were increased significantly, and inversely correlated with *FBLN5* gene expression. FBLN5 degradation products of 52.8 and 41.3 kDa were increased substantially in EOC. We identified two candidate proteases (serine elastase and MMP-7, but not MMP-9) that cleave FBLN5. MMP-7, but not neutrophil elastase, gene expression was increased dramatically in EOC. Recombinant FBLN5 significantly inhibited adhesion of EOC cells to both laminin and collagen I. Finally, using immunohistochemistry, we found immunoreactive FBLN5 within tumor macrophages throughout human EOC tumors. This work indicates that FBLN5 is degraded in EOC most likely by proteases enriched in macrophages of the tumor microenvironment. Proteolysis of FBLN5 serves as a mechanism to promote cell adhesion and local metastasis of ovarian cancer cells. Promotion of a stable ECM with intact FBLN5 in the tumor matrix may serve as a novel therapeutic adjunct to prevent spread of ovarian cancer.

## INTRODUCTION

Ovarian cancer is the second most common gynecologic malignancy in the US, accounting for approximately 22,000 new cases per year [1]. It is, however, the deadliest gynecologic malignancy, due, in part, to the fact that 75% of new diagnoses are discovered after the cancer has metastasized [2]. The steps required for the development of metastases represent complex interactions between tumor cells and the host microenvironment that include adhesion of cancer cells to distant sites and infiltration through the extracellular matrix (ECM).

The fibulin family consists of seven ECM proteins characterized by tandem arrays of epidermal growth factor-like domains and a C-terminal fibulin-like module [3]. Altered expression and function of fibulin 5 has been implicated in several human cancers [4–8]. In general, it is considered a suppressor of tumor angiogenesis and a potential tumor suppressor, in part through its inhibition of the gelatinases, MMP2 and MMP9 [4, 5, 9]. Fibulin-5 is a 448 amino acid, 66 kDa, glycoprotein secreted by smooth muscle cells, fibroblasts and vascular endothelial cells. It has been shown to be prominent in blood vessel walls and in the basement membrane underlying epithelial cells [10]. Functionally, it is a crucial protein in the formation of elastic fibers as it tethers tropoelastin to microfibrils where tropoelastin is cross-linked by lysyl oxidases to form mature elastic fibers [11]. Additionally, it mediates endothelial cell adhesion through its integrin binding domain, and has been shown to be dramatically induced in vascular endothelial and smooth muscle cells in response to injury [12]. On the other hand, FBLN5 antagonizes VEGF signaling resulting in diminution of VEGF-induced proliferation and migration of endothelial cells [13].

Recently, we showed that the RGD domain of FBLN5 was not necessary for FBLN5-mediated elastogenesis but was crucial for inhibition of MMP-9 in the female reproductive tract [14]. The gelatinases, MMP2 and MMP9, are both released in a pro-form that must be converted to the active form to function. Activated gelatinases have the ability to degrade type IV collagen in the basement membrane, an event considered key in tumor cell invasion and metastasis. Studies of human epithelial ovarian cancer (EOC) suggest that overexpression of MMP9 in ovarian cancer stroma is a significant predictor of shortened disease-specific survival [15].

Here, we explored the ECM in human EOC compared with benign ovarian tissue. At the protein level, 66 kDa FBLN5 protein was identified as two additional immunoreactive species, 53 kDa and 41.2 kDa in malignant tissues which were immunoreactive in tumor macrophages, not cancer cells or stroma. Two proteases enriched in macrophages (serine elastase and MMP-7) degraded FBLN5 *in vitro*. Finally, we showed that FBLN5 degradation may play an important pathologic role in EOC because full length FBLN5 inhibited cancer cell adhesion to select basement membrane proteins laminin and type I collagen. Taken together, the data suggest that FBLN5 is degraded in epithelial ovarian cancer possibly by tumor macrophages that support tumor growth by degrading the matricellular protein thereby leading to increased angiogenesis and upregulation of MMP-9 to further support the invasive phenotype of EOC.

## RESULTS

### Fibulin genes are downregulated in ovarian cancer tissues relative to benign ovarian tissue

**Figure 1:**
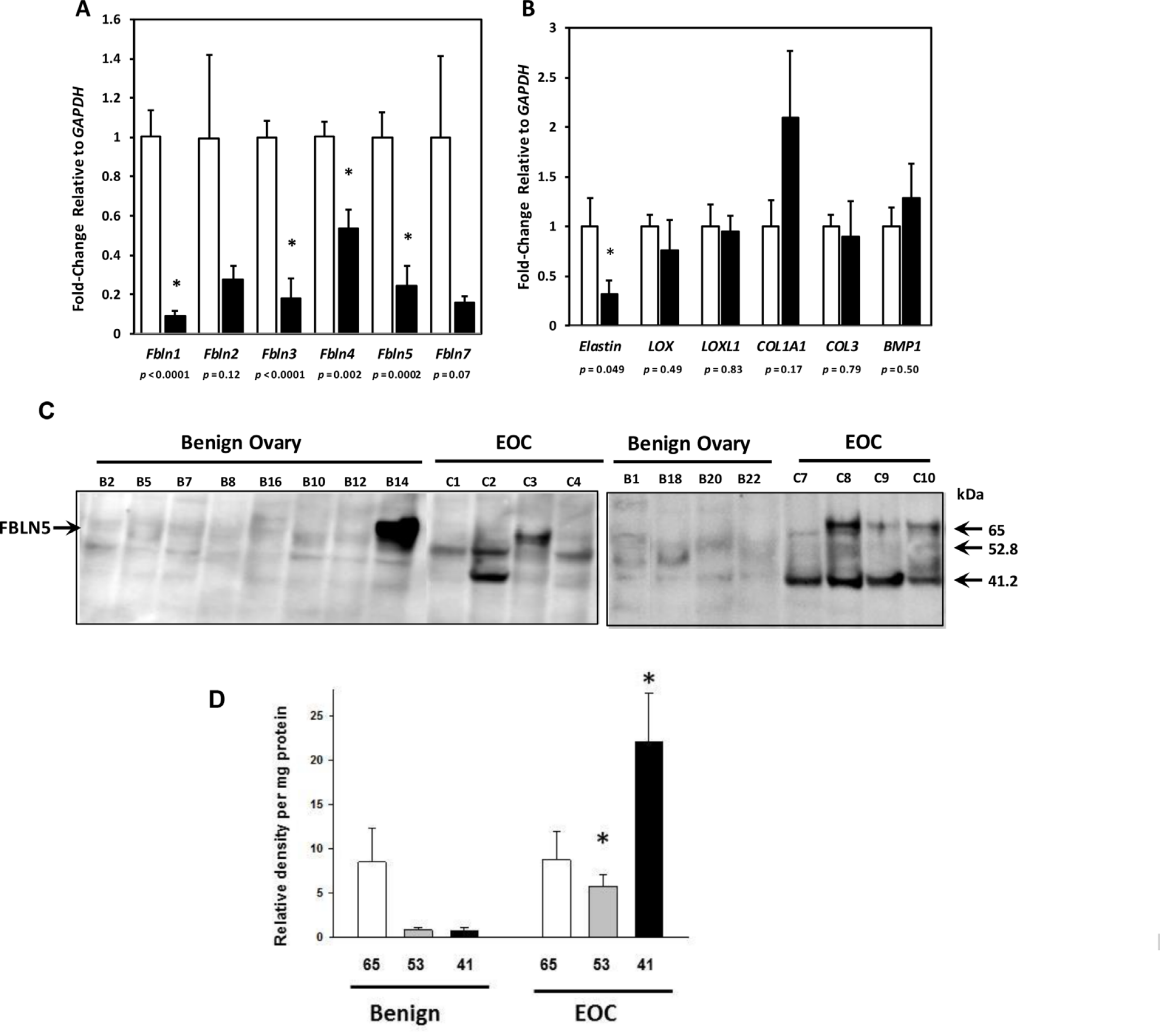
Gene expression of Fibulin family members and matrix proteins in epithelial ovarian cancer (EOC) (**A**) Relative levels of *FBLN* mRNA were quantified in ovarian tissue from benign (open bar, *n* = 12) and EOC (solid bar, serous, *n* = 8; mucinous, *n* = 3). (**B**) Relative levels of mRNA encoding other matrix proteins (*Elastin*, tropoelastin; *LOX,* lysyl oxidase; *LOXL1*, lysyl oxidase like 1; *COL1A1,* collagen type Ia; *COL3,* collagen type 3, and BMP1, bone morphogenic protein-1) in ovarian tissue from benign (open bar, *n* = 12) and EOC (solid bar, serous, *n* = 8; mucinous, *n* = 3). Data represent mean ± SD. ^*^P < 0.05 compared with benign. (**C**) Fibulin-5 expression in matricellular extracts from benign ovary and EOC. Representative immunoblots of FBLN5 in urea extracts from benign ovary and EOC. Numbers identify each tissue. (**D**) Relative density units of 65-, 53-, and 41- kDa FBLN5 in benign ovarian tissue (*n* = 12) and EOC (*n* = 11). Data represent mean ± SEM normalized to total urea-extracted protein quantified on paired Coomassie blots. ^*^P < 0.05 compared with benign.

To determine if fibulin genes are differentially regulated in ovarian cancer tissue relative to benign ovarian tissue, RNA was extracted from tissue samples and relative mRNA levels of *FBLN1*, *FBLN2*, *FBLN3*, *FBLN4*, *FBLN5* and *FBLN7* were quantified using qPCR (Figure 1A)*. FBLN1* (approximately 10-fold), *FBLN3* (~5-fold), *FLBN5* (~5-fold) and *FBLN4* (~2-fold) were downregulated significantly. Although not statistically significant, there was a trend toward downregulation of *FBLN2* and *FBLN7* in cancer tissue relative to benign. We considered the possibility that matrix proteins may be globally downregulated in EOC simply due to a disproportionate number of ovarian epithelial cells relative to matrix-secreting stromal fibroblasts. Thus, we examined relative mRNA expression of additional ECM proteins: tropoelastin, two collagen and elastin cross-linking enzymes (*LOX* and *LOXL1*), two fibrillary collagen genes (*COL1A1* and *COL3*) and procollagen peptidase (bone morphogenic protein-1, *BMP1*). Tropoelastin mRNA was downregulated 2-fold in cancer tissue relative to benign (Figure 1B). Expression of the lysyl oxidase family members, *LOX* and *LOXL1*, was similar between malignant and benign ovarian tissues. The genes encoding major protein components of type I and type III collagens, *COL1A1* and *COL3*, as well as *BMP1* (a metalloprotease that cleaves the c-terminus of procollagens I, II and III) were also similar between the two tissue types (Figure 1B). Taken together, these results suggest that although many genes involved in collagen and elastin synthesis were normally expressed, specific fibulins and tropoelastin are downregulated in ovarian cancer.

Little is known regarding the role of most fibulin family members in EOC. Several lines of evidence indicate that FBLN5 may mediate cell adhesion through its integrin binding domain [3, 16–18] and may modulate cell migration [7, 12, 19–22]. Hence, we focused on FBLN5 and conducted immunoblot analysis of urea-extracted matrix from benign and malignant ovarian tissue. Although not detectable in soluble fractions (not shown), FBLN5 was expressed in the matrix (i.e., urea-extracts) (Figure 1C, 1D). Low levels of immunoreactive FBLN5 were expressed in benign ovarian tissue (Figure 1C). Interestingly, however, immunoreactive proteins of 65 kDa (full-length FBLN5) were expressed in some, but not all, EOCs. Rather, immunoreactive intensities of 52.8 and 41.2 kDa proteins were increased in EOC relative to benign ovarian tissue. The antibody used in this study was a polyclonal FBLN5 antibody raised against full length rat FBLN5 that recognizes the human protein [23]. Blots developed under identical conditions but without the primary FBLN5 antibody did not reveal bands of 52 or 41 kDa. We therefore considered these immunoreactive proteins as degraded FBLN5. The 41 kDa protein was virtually absent in benign ovary but highly expressed in 6 of 11 EOCs (55%, Figure 1D).

### FBLN5 is not expressed in EOC cancer cells in culture

**Figure 2:**
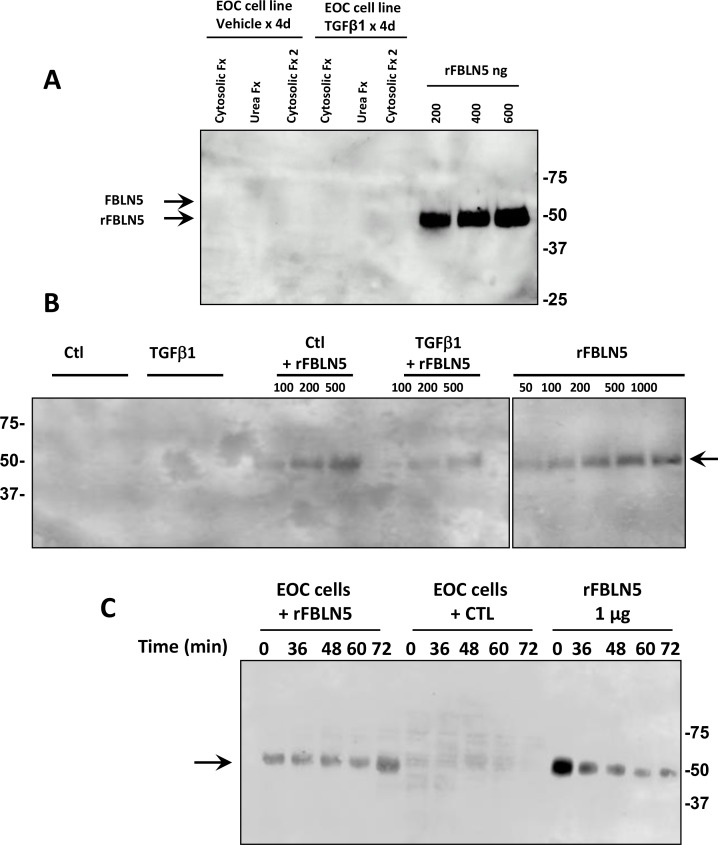
Fibulin-5 is not expressed and not metabolized by EOC cell lines (**A**) Immunoblot analysis of FBLN5 in cytosolic and urea extracts from EOC cell line 5020 treated with vehicle or TGFβ1 (5 μg/ml) for 4 d. Results are representative of 6 cell lines. Cytosolic Fx 2 indicates supernatant of second homogenization of tissue pellet in cytosolic buffer. Arrows denote migration of endogenous glycosylated FBLN5 (65 kDa) (which is absent in these cells) or purified rFBLN-5 (51 kDa). (**B**) Culture media recovered from EOC cell line 5020 [24] incubated with vehicle (Ctl) or TGFβ1 (5 μg/ml) for 48 h ± rFBLN5. rFBLN5 was recovered intact with no proteolytic fragments. (**C**) EOC cell extracts (30 μg) incubated with or without rFBLN5 (1 μg) as a function of time.

The finding of increased degradation of FBLN5 in EOC tumors led us to consider use of EOC cell lines to investigate regulation of FBLN5. Cytosolic and urea extracts from two commonly used EOC cell lines, HTB77 (SKOV3) and HTB161 (OVCAR3), together with 5 cell lines (Table 1) generated at UTSW [24] were evaluated. FBLN5 was not expressed in any EOC cell line at mRNA or protein level (Figure 2A). Further, TGFβ1, a known inducer of EMT and FBLN5 gene expression, did not increase FBLN5 in these cells (Figure 2B). Culture media recovered from EOC cell lines treated with vehicle (Ctl) or TGFβ1 (5 μg/ml) for 48 h did not proteolyze FBLN5 (Figure 2B), and cancer cell extracts did not metabolize FBLN5 (Figure 2C). These data indicate that (i) FBLN5 gene expression is absent in malignant ovarian epithelial cells but may be expressed in other cell types within the tumor, and (ii) epithelial ovarian cancer cells do not metabolize FBLN5.

**Table 1:** Clinical information regarding cell lines used in this study

Cell line	Ascites (ml)	Site of disease	Stage of disease	Histology	Grade	Race
HCC 5011	3000	Ovary	IIIC	Serous	2–3	White
HCC 5019	1500	Ovary	IV	Serous Cystadenocarcinoma	3	Black
HCC 5020	1000	Ovary	IV	Papillary Serous Cystadenocarcinoma	3	Black
HCC 5022	1500	Ovary	IIIC	Papillary Serous	3	Hispanic
HCC 5023	2500	Ovary		Serous Adenocarcinoma	3	Asian

### MMP9 is upregulated in ovarian cancer whereas TIMP2 is downregulated

**Figure 3:**
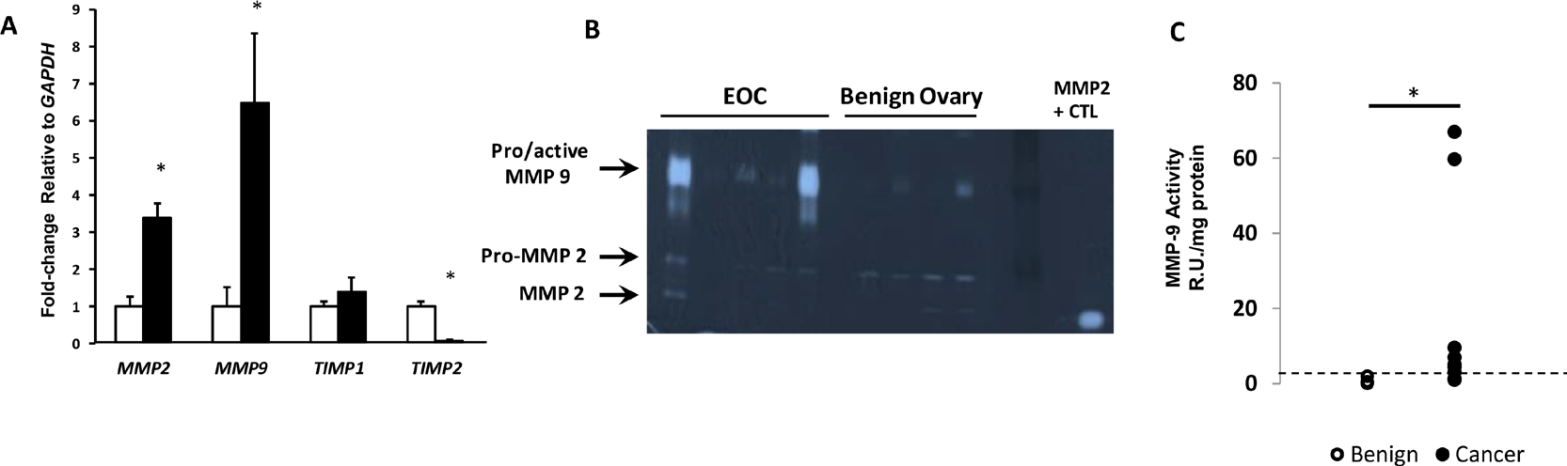
Expression of MMP2 and MMP9 in EOC (**A**) Relative levels of *MMP2*, *MMP9*, and MMP inhibitors (*TIMP1* and *TIMP2*) mRNA were quantified in ovarian tissue from benign (open bar, *n* = 12) and EOC (solid bar, *n* = 11). Data represent mean ± SEM. ^*^P < 0.05 compared with benign. (**B**) Representative gelatin zymogram of MMP-9 and MMP-2 activity in benign ovary and EOC. (**C**) Relative units of MMP-9 activity per mg protein extracts from benign (*n* = 12) and EOC (*n* = 10). Dashed line indicates 95% confidence interval of benign tissues. ^*^P < 0.03, Fisher's exact.

Previously, we showed that FBLN5 downregulated vaginal MMP-9 through its integrin binding domain [14]. This result was in agreement with studies in which fibulin 5 inhibited induction of MMP9 in mouse mammary adenocarcinoma [4]. Nonetheless, this effect appeared to be tissue-specific because loss of FBLN5 does not lead to upregulation of MMP-9 in the vasculature or lung [14]. To determine, therefore, whether downregulation of FBLN5 in EOC was associated with upregulation of MMP-9, expression of the gelatinases, *MMP2* and *MMP9*, was quantified in benign and malignant ovarian tissue. *MMP2* was upregulated 3-fold and *MMP-9* 6.5-fold in ovarian cancer (Figure 3A). We also examined their inhibitors, *TIMP1* and *TIMP2*. *TIMP2,* but not *TIMP1,* gene expression was downregulated 15-fold in malignant tissues. The results indicate that downregulation of FBLN5 in ovarian cancer was accompanied by dramatic induction of *MMP-9* and loss of its inhibitor, *TIMP2*.

As mRNA transcripts do not always reflect *in vivo* enzyme activity, quantitative gelatin zymography was conducted to analyze the relative activity of MMP9 in benign and malignant ovarian tissues. Although pro- and active forms of MMP9 are difficult to separate, in benign tissues, MMP-9 was either nondetectable or weak whereas both pro- and active MMP-2 were detected. In EOC tissue, MMP-9 was increased significantly (Figure 3B). Using the 95% confidence interval for benign ovary as the upper limit for normal, MMP-9 was increased in 2 of 12 benign samples, and 7 of 10 cancer samples. MMP9 activity was increased 3- to 9-fold in 5 and 60- to 65-fold in 2 of 10 cancer samples (Figure 3C, *p* = 0.03, Fisher's Exact Test).

**Figure 4:**
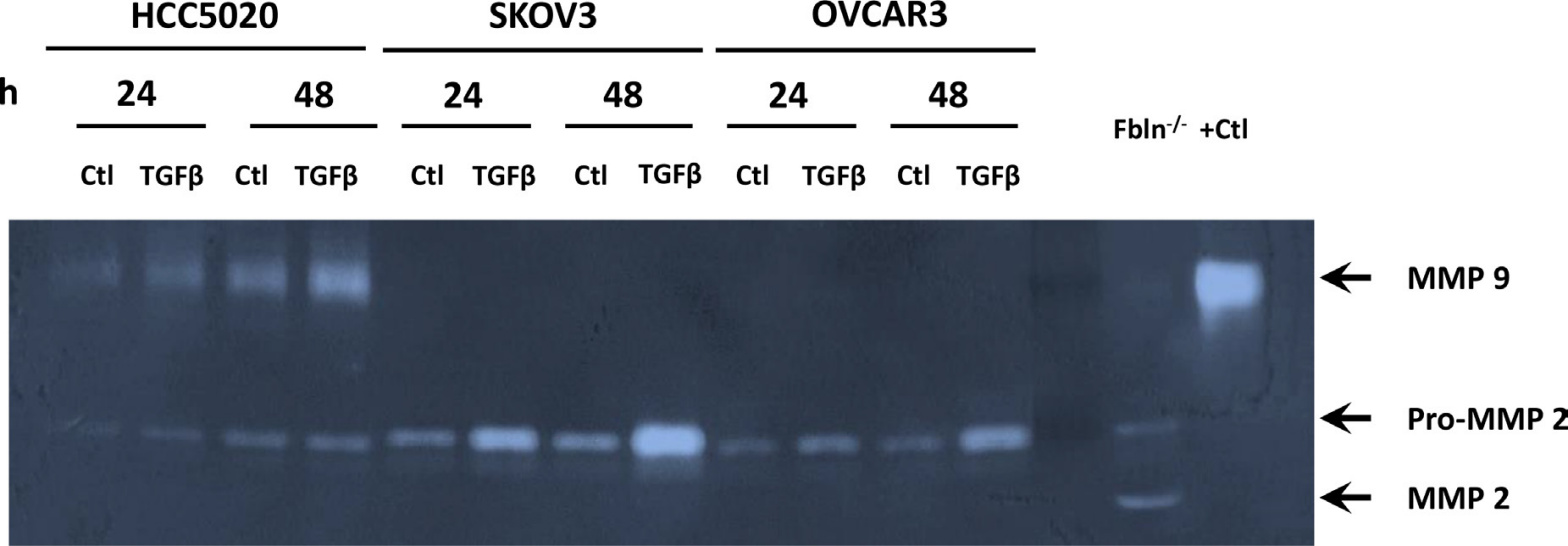
MMP-9 activity is increased in recently discovered ovarian cancer cell lines, but not HTB77 (SKOV3) or HTB161 (OVCAR3) Representative zymogram of media from EOC cell lines cultured with vehicle (Ctl) or TGFβ1 (5 μg/ml) for 24–48 h. Positive controls were vaginal tissue from *Fbln5* null mice or purified MMP-9.

Next, we assessed MMP-2 and MMP-9 activity in ovarian cancer cell lines (Figure 4). Interestingly, although proMMP-2 was expressed and regulated by TGFβ1, MMP-9 activity was not expressed in SKOV3 or OVCAR3 and was not induced by TGFβ1. In contrast, MMP9 activity was readily detectable in 4 of 5 newly-derived lines (passage 22–25) [24], and was induced by TGFβ1 (e.g., HCC5020, Figure 4).

### Candidate proteases to degrade fibulin-5

**Figure 5:**
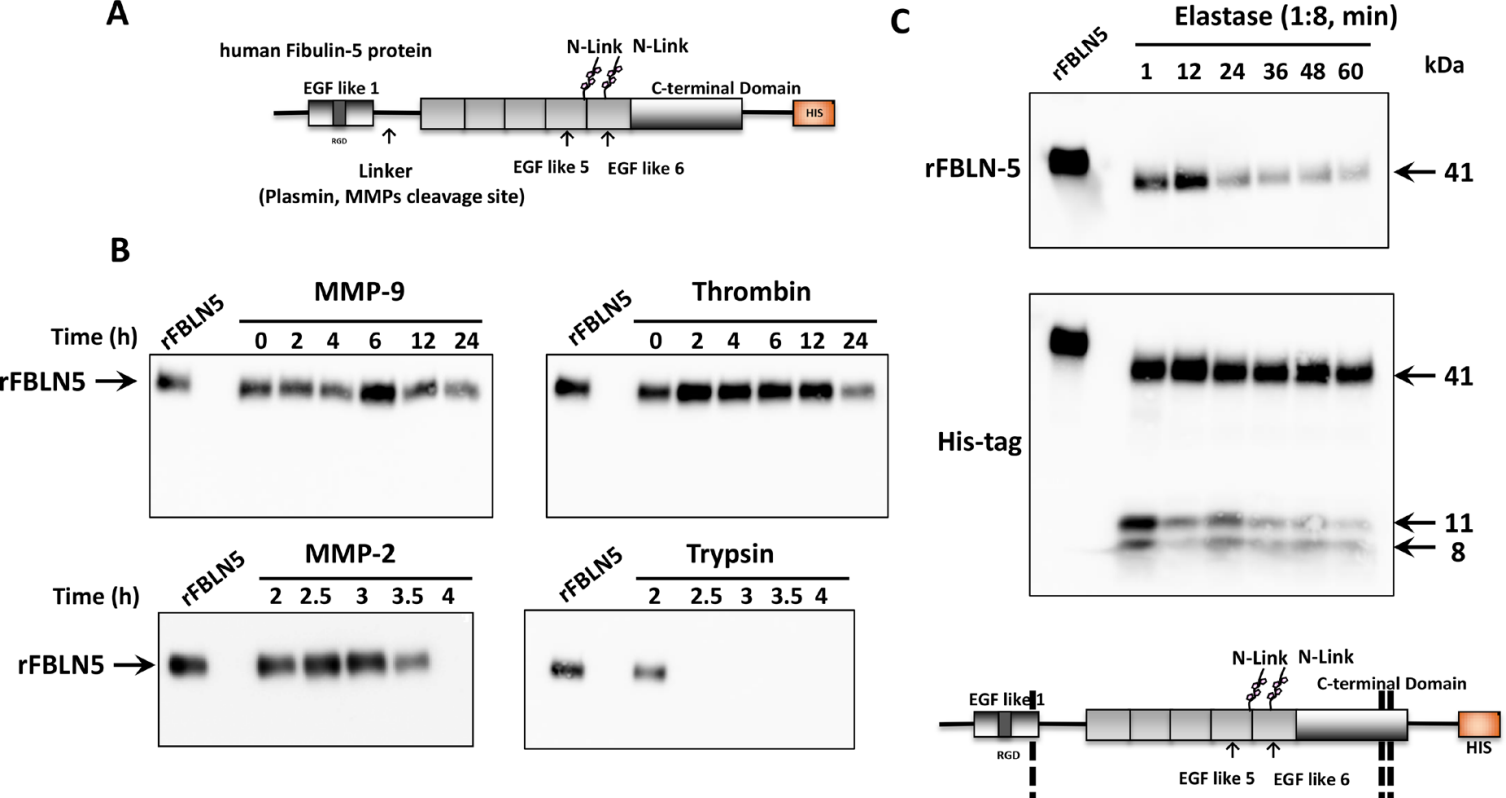
*In vitro* FBLN5 protease assays (**A**) Diagram of recombinant human FBLN5. The amino terminus containing the RGD domain is followed by a linker region, previously described as a cleavage site for plasmin or MMPs. Glycosylated sites are noted and the C-terminal HIS tag is illustrated. (**B**) Purified MMP-9, MMP-2, thrombin, and trypsin were incubated with rFBLN5 as a function of time. Products were visualized using immunoblot analysis and a monoclonal antibody to human FBLN5. (**C**) Purified elastase was incubated with rFBLN5 (1:8 molar ratio) as a function of time. Thereafter, products were visualized using immunoblot analysis and antibodies to FBLN5 (upper blot) or the HIS tag (lower blot). Approximate cleavage sites are noted in the diagram.

**Figure 6:**
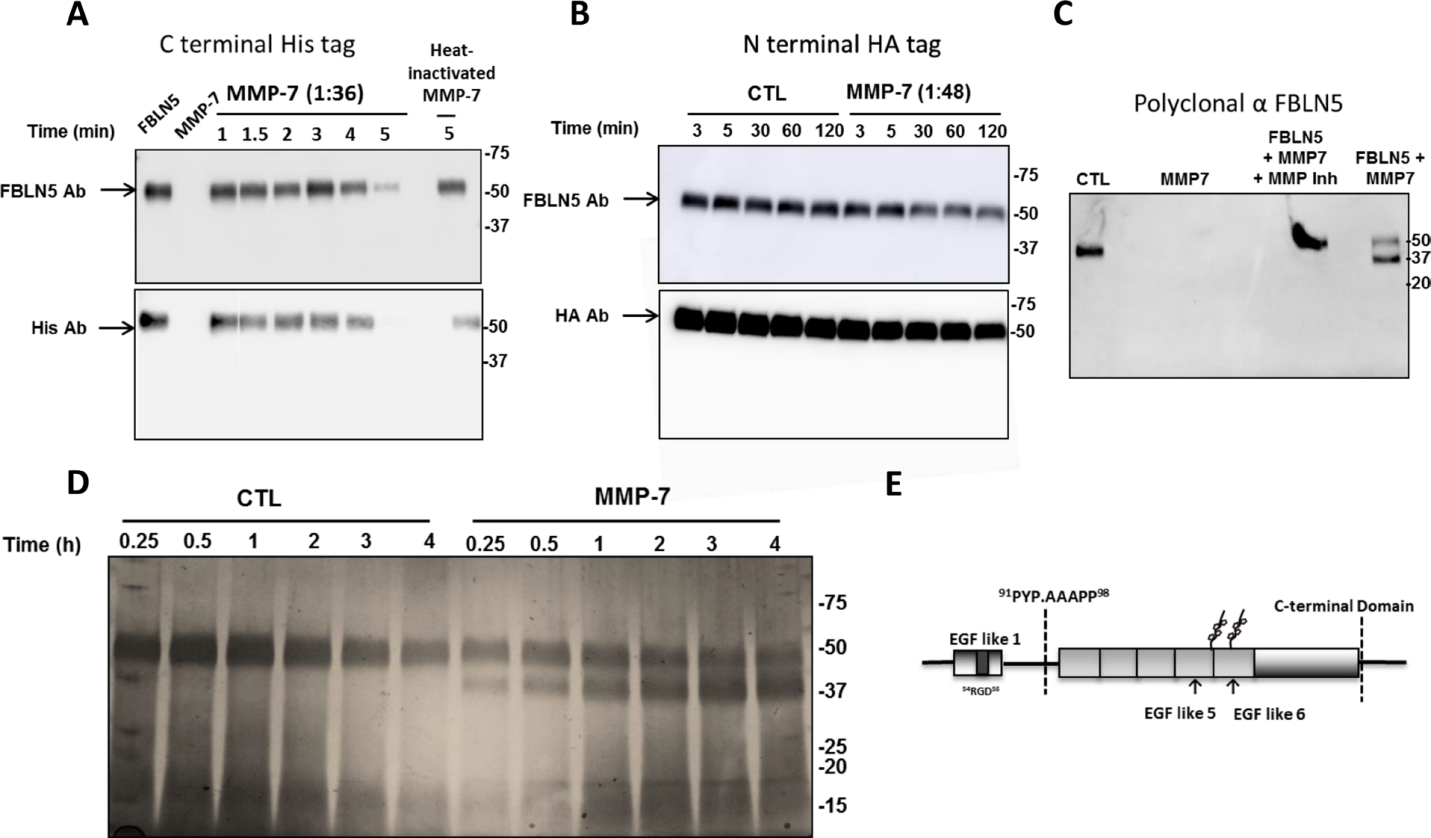
Effect of MMP-7 on degradation of FBLN5 *in vitro* (**A**) Purified MMP-7 (1:36 molar ratio) was incubated with rFBLN5 as a function of time. Thereafter, products were visualized using immunoblot analysis and antibodies to FBLN5 or HIS tag. (**B**) Diluted MMP-7 was incubated as a function of time (in minutes) with rFBLN5 with an amino terminus HA tag. (**C**) FBLN5 (200 ng), MMP-7 (1:36), or FBLN5+MMP7 ± actinonin (a general MMP inhibitor) was incubated at 37° C for 5 min. Thereafter, the proteins were transferred an incubated with a polyclonal antibody to FBLN5. (**D**) Silver stain of FBLN5 incubated for 4 min with or without MMP-7. (**E**) Diagramatic sites of cleavage of MMP-7. Sequence of cut site is shown.

We considered possible proteases that degrade FBLN5 and would thereby prevent the tumor suppressive functions of FBLN5. A candidate protease approach was taken. Recombinant human FBLN5 protein (Gln^24^-Phe^448^) with a carboxy-terminal poly-histidine tag was incubated with various candidate proteases, and immunoblotting was performed with antibodies to FBLN5 or an anti-His antibody to detect the C-terminal product as indicated in Figures 5–6. Incubation of rFBLN5 (Figure 5A) with MMP9 resulted in no appreciable degradation of rFBLN5 for up to 24 h (Figure 5B). In contrast, MMP2 resulted in complete degradation of rFBLN5 within 4 h. Degradation products similar to those found in EOC tissue samples, however, were not visualized. Likewise, the serine protease, thrombin, had a modest effect in degrading rFBLN5 by 24 h. As expected, trypsin rapidly and completely degraded rFBLN5. For these four proteases, HIS-tag antibodies identified products identical to those of FBLN5 antibodies with no immunoreactive degradative products (not shown).

Next, we considered the possibility that elastase, a serine protease known to be expressed in pancreas [25, 26] and neutrophils [27], may mediate degradation of FBLN5. To interpret the results of this experiment, it should be emphasized that, unlike endogenous FBLN5, rFBLN5 is not glycosylated and the first 24 amino acids are deleted. Therefore rFBLN5 migrates at 50.6 kDa rather than 65–66 kDa on SDS gels. Within one min, rFBLN5 was converted to a 41–43 kDa product and two 11 and 8 kDa proteins immunoreactive for the HIS tag (Figure 5C). Both products decreased as a function of time. The MEROPS database (http://merops.sanger.ac.uk) reveals 20 potential elastase cleavage sites. Our results suggest that elastase proteolyzes rFBLN5 at three sites. The 41 kDa product indicates a cut site at the amino terminus consistent with the previously described linker region. The smaller products indicate additional cut sites at the C-terminus (Figure 5C).

Finally, MMP7, a matrix metalloproteinase implicated in metastasis of other malignancies [5, 28–30] was considered. Incubation of 200 ng rFBLN5 with MMP7 at a molar ratio of 1:1.8 led to complete degradation of FBLN5 within seconds. Hence, the enzyme was diluted (Figure 6A) demonstrating rapid proteolysis of FBLN5 within 5 min which was rescued by heat inactivation of the enzyme. Degradation products were not visualized with the monoclonal FBLN5 antibody (Figure 6B). The sites of cleavage, therefore, were not clear and investigated further. rFBLN5 tagged at the amino terminus with HA was incubated with MMP-7 and products visualized with HA antibodies and the monoclonal antibody to FBLN5 (Figure 6B). MMP-7 degraded FBLN5, but HA antibodies did not detect the cleaved product. Polyclonal FBLN5 antibodies and silver staining revealed a 37 kDa degraded product (Figure 6C, 6D). Full length and 37 kDa bands were excised and analyzed by mass spectrometry. The 37 kDa protein was identified as amino acid residues A^94^ –Phe^448^. Thus, the cleavage site was within the linker region in the insertion site of the first calcium binding EGF-like repeat (Figure 6E). Together with the first 24 amino acids *in vivo*, the protein would represent a 41 kDa product. The results indicate that MMP-7 also cleaves the C-terminal His tag.

### MMP7 expression in malignant and benign ovarian tissues

**Figure 7:**
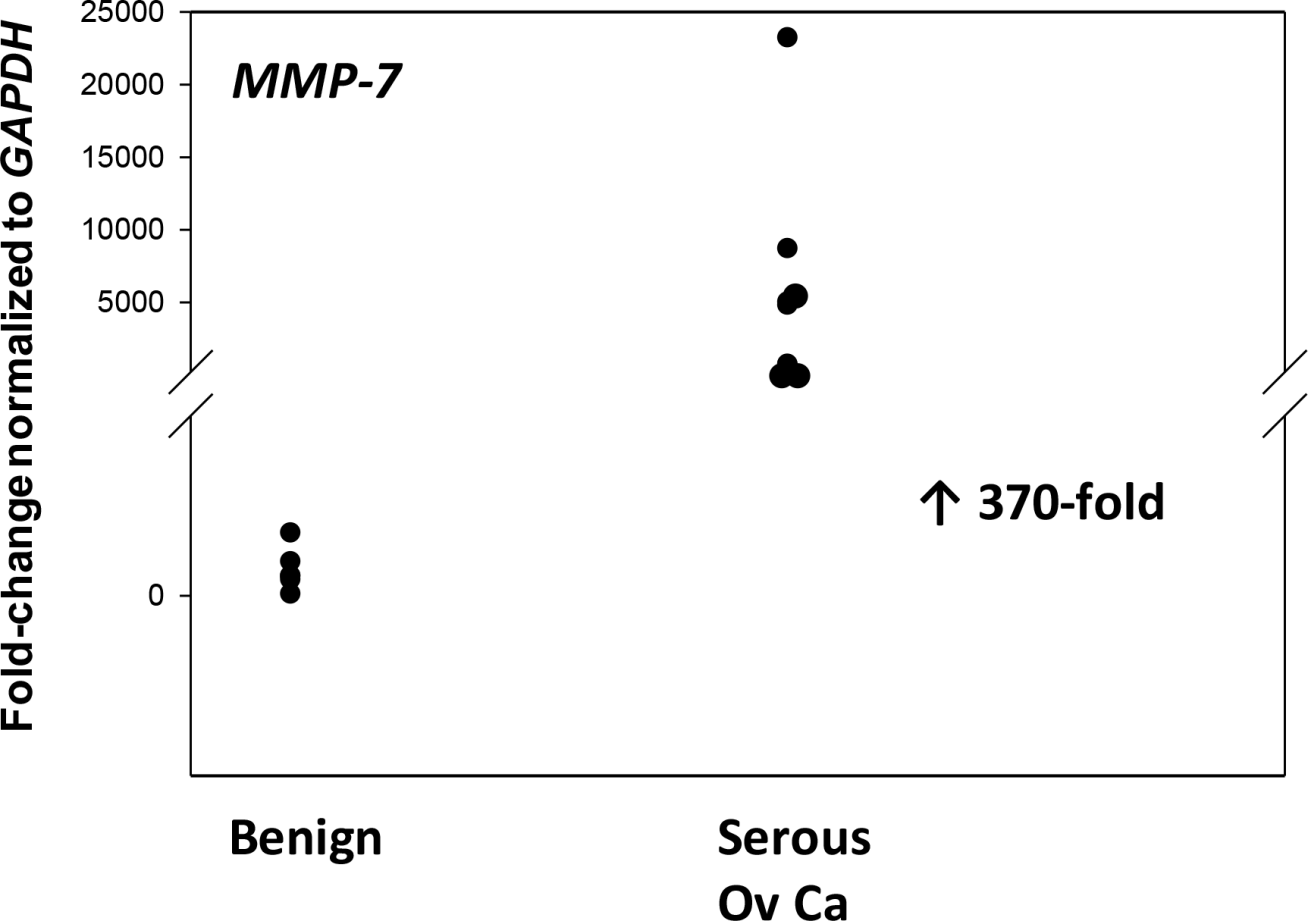
MMP-7 gene expression is upregulated in advanced serous EOC Relative levels of MMP-7 mRNA were quantified in ovarian tissue from benign (*n* = 12) and EOC (serous, *n* = 8). *P <* 0.001.

Identification of two proteases with unique cut sites for FBLN5 led us to evaluate MMP7 and elastase gene expression in benign and malignant ovarian tissues. Differential expression of *MMP-7* was highly significant in which *MMP7* expression was barely detectable by qPCR in benign ovarian tissues (Cq = ~30, Figure 7) but highly expressed in cancer (Cq = 25, Figure 7) representing striking increases of 370-fold. Neutrophil elastase, on the other hand, was poorly expressed in both tissue types.

### FBLN5 is localized to human tumor macrophages

**Figure 8:**
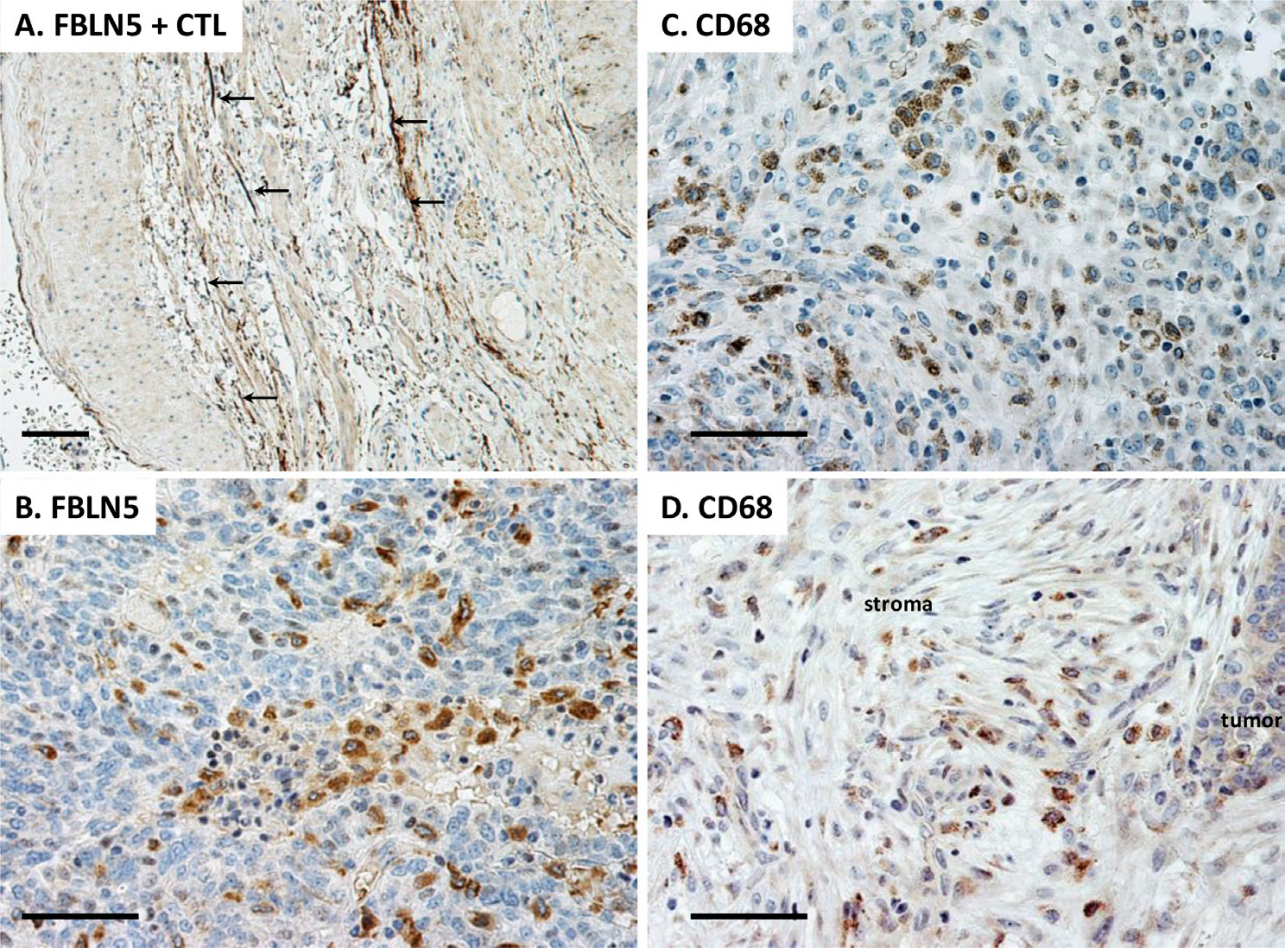
Immunolocalization of FBLN5 in EOC Sections were stained with either FBLN5 antibodies (**A**, **B**) or the macrophage marker CD68 (**C**, **D**). The positive control is shown as large arteries from the utero-ovarian vessels. FBLN5 (arrows) is localized to elastic fibers of blood vessels. Tumor macrophages, but not malignant epithelial cells, are positive for FBLN5 (**B**). Macrophages are highlighted by CD68 staining in EOC (**C**) and stroma of the EOC (**D**). Images represent reproducible and consistent results from 3 high grade serous tumors. Immunostaining without primary antibody is negative. Bar = 50 μm.

Having established increased immunoreactive degradative products of FBLN5 in EOC, next we used immunohistochemistry to localize the protein in these human epithelial ovarian cancers (Figure 8). Large utero-ovarian arteries were used as the positive control in which FBLN5 was clearly positive in the extracellular matrix outlining elastic fibers of the vessel wall (Figure 8A). FBLN5 was not observed in blood vessels of ovarian tumors. Rather, the protein was localized to the cytoplasm and granules of tumor macrophages (Figure 8B). Stromal fibroblasts and tumor cells were negative for FBLN5. CD68+ tumor macrophages were distributed throughout the tumor (Figure 8C) and the stromal compartment (Figure 8D), both of which were positive for FBLN5. FBLN5 was absent in macrophages of benign ovary.

### Functional significance of FBLN5 in EOC

**Figure 9:**
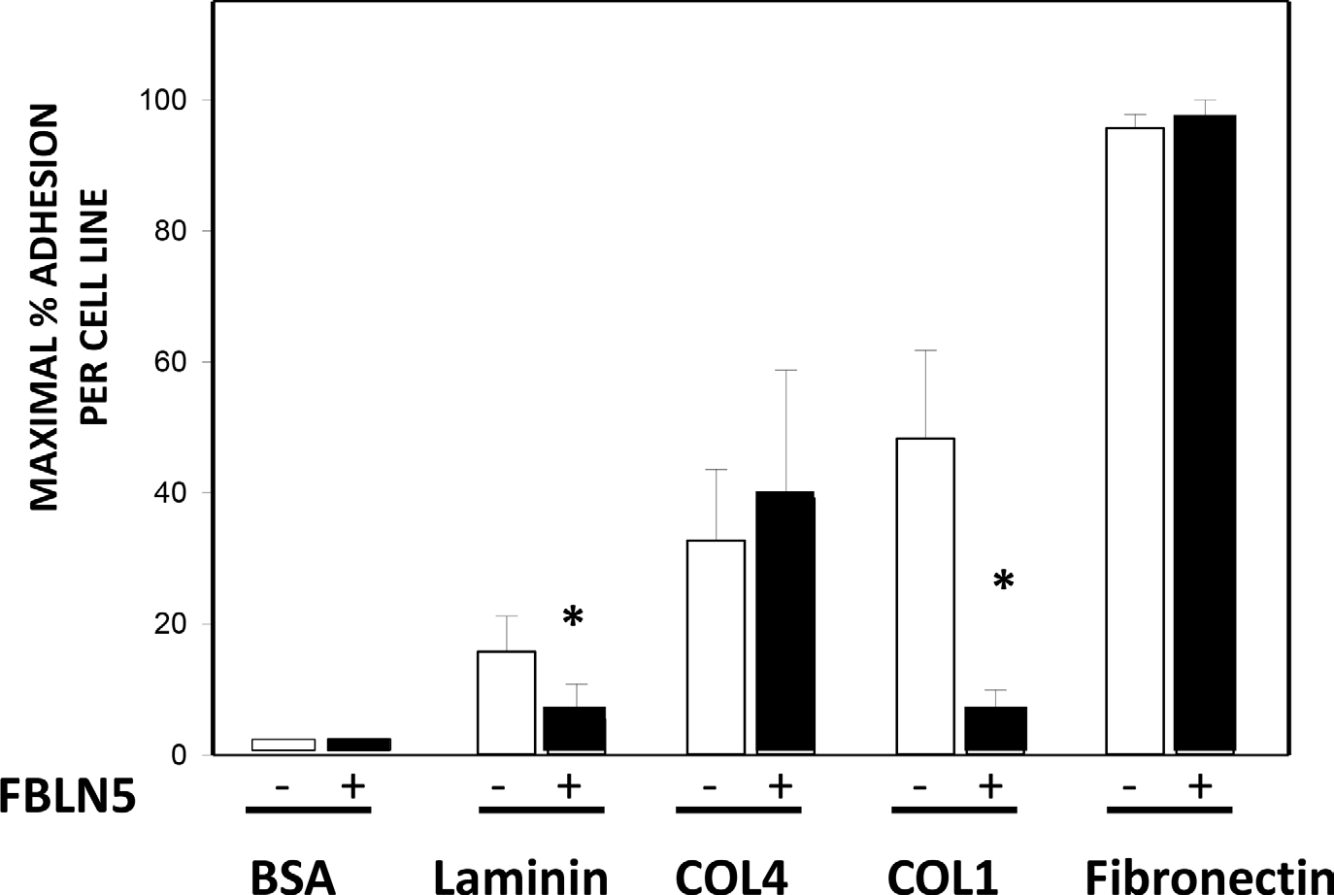
Effect of FBLN5 on EOC cell adhesion Cell adhesion assays were conducted with 3 different EOC cell lines (HCC 5019, 5020, and 5022) on various substrates. Adhesion to fibronectin was considered maximal and maximal adhesion for each cell line was considered 100%. Bars represent mean ± SEM of 3 cell lines analyzed in triplicate. ^*^P < 0.05 compared with matrix without FBLN5.

Next, we sought to explore the potential function of FBLN5, and more specifically, the physiologic consequence of its degradation in malignant tissues. To answer this question, we turned to cell culture using several ovarian cancer cell lines produced at our institution [24]. We hypothesized that intact FBLN5 may either inhibit tumor cell-derived MMP-9 or inhibit malignant cell adhesion to native ECM components and thereby protect from local spread of the disease. Degradation of FBLN5 would thereby increase MMP-9 protease activity or facilitate ovarian cancer cell adhesion to matrix substrates. Interestingly, although incubation of EOC cells in culture with rFBLN5 did not inhibit tumor cell MMP-9 (not shown), rFBLN5 inhibited adhesion of EOC cells to specific matrix components (Figure 9). Cells from three ovarian cancer cell lines (HCC 5019, 5020, and 5022) were incubated in tissue culture wells pre-coated with a variety of ECM proteins (laminin, collagen type IV, collagen type I and fibronectin) ± rFBLN5. Bovine serum albumin (BSA) was used as a protein control. Ovarian cancer cells adhered readily to wells coated with fibronectin, regardless of treatment with rFBLN5 (Figure 9). Adherence to other ECM proteins was computed relative to fibronectin. FBLN5 significantly inhibited EOC cell adhesion to both laminin and collagen I. One physiologic function of FBLN5 is likely to decrease the ability of malignant epithelial ovarian cancer cells to adhere to ECM proteins laminin and type I collagen.

## DISCUSSION

Tumor metastasis beyond the organ of origin is the leading cause of cancer-related mortality. The steps leading to metastasis include (1) local invasion of tumor through a basement membrane, (2) spread of tumor to a distant site (*via* hematogenous or lymphatic spread, or by direct extension or through peritoneal fluid) and (3) implantation, invasion and proliferation at the distant site. In the present study, we explore the role of the matricellular protein, FBLN5, in the final step of metastasis.

### Physiologic significance of reduced expression of FBLN5 in cancer

FBLN5 has been studied in the context of several epithelial malignancies including breast [4], lung [5], pancreas [6], bladder [7] and prostate [8]. In general, it is thought to represent a tumor suppressing protein, and is often shown to decrease invasion and metastatic colonization of these tumors. Its role in metastatic ovarian cancer is less well elucidated. In agreement with the current study, Heo *et al.* showed that FBLN5 protein was decreased in EOC relative to 3 benign ovarian cystadenomas and that overexpression of FBLN5 in SKOV3 cells inhibited cell migration and invasion [31]. In our study, we first sought to compare the RNA expression levels of different fibulin proteins in both benign and malignant ovarian tissues. In our samples, *Fbln1, −3, −4* and *-5* were significantly downregulated in epithelial ovarian cancer (EOC) relative to benign ovarian tissue. This downregulation of *FBLN5* in EOC is consistent with downregulation described in other epithelial malignancies [4–8]. Messenger RNA levels of genes encoding many other matrix components were similar between benign and malignant ovarian tissue. Although far from a comprehensive investigation regarding ECM regulation, overall the data suggest that specific fibulins, and not other genes involved in collagen synthesis, are downregulated in EOC.

Two relevant studies demonstrated that reduced levels of FBLN5 result in increased propensity for metastatic spread. Moller, *et al.*, demonstrated that expression of *Fbln5* was decreased in fibroblasts incubated with tumor-conditioned media from mouse mammary adenocarcinoma cell lines compared with fibroblasts cultured in standard media. Additionally, injection of cell lines modified to overexpress *Fbln5* into mice resulted in fewer metastatic deposits than unaltered cancer cell lines [4]. Similarly Hu, *et al.*, reported that FBLN5 protein expression was decreased in human urothelial carcinomas compared with benign bladder tissue. Transfection of FBLN5 in a bladder cancer cell line resulted in decreased growth rates, cell invasion and cell migration compared with non-transfected and empty vector-transfected controls [7]. Interestingly, transfection of Fbln5 into our EOC cell lines resulted in cell death even at low levels of transfection reagents (data not shown). These results, taken together with several lines of investigation [4, 7], led us to suggest that decreased *Fbln5* in EOC may result in increased invasiveness and metastasis.

### Relationship between MMPs and FBLN5

In several studies, expression of FBLN5 was related to expression of the gelatinases, MMP2 and MMP9. In experiments by Moller [4], conditioned media from mammary adenocarcinoma cell lines significantly increased expression of MMP9 in fibroblasts. These results were countered by overexpression of FBLN5. Additionally, by culturing their cells with an MMP2/9 selective inhibitor, they showed inability of these cells to invade an extra-cellular matrix model [4]. Our previous work extended these observations. Using a knock-in model of FBLN5 in which the integrin binding RGD domain was mutated to RGE, we demonstrated that FBLN5-mediated suppression of MMP-9 was cell-specific and mediated through its RGD domain [14]. It was not surprising, therefore, that FBLN5 suppresses MMP-9 in some cells, but not in our malignant EOC cell lines. Nonetheless, this result does not rule out the possibility that FBLN5 may suppress MMP-9 generated in tumor stroma or macrophages. Huang, *et al.*, reported that host-derived MMP9 expression, likely from tumor-infiltrating macrophages, played a critical role in angiogenesis and progressive growth of human ovarian tumors in mice [32]. In contrast, Shibata, *et al.*, concluded that host peritoneal/mesothelial cells secrete an unidentified factor that induces MMP9 in human EOC cell lines [33]. In our studies, degradation of FBLN5 appeared to be independent of tumor cells in culture or tissue extracts. It is possible that tumor macrophages, or peritoneal cells, may mediate FBLN5 degradation in EOC tumors. Further, the unidentified factor described by Shibata *et al..* may represent a protease that degrades FBLN5 in EOC.

The clinical implications of increased MMP9 have been independently demonstrated by Kamat and Sillanpaa [15]. Both showed that stromal expression of MMP9 resulted in shortened disease-specific survival in patients with EOC [15, 34]. Finally, Yang, *et al.*, demonstrated improved survival in a murine orthotopic model of disseminated ovarian cancer with treatment with a conditionally replicating adenovirus expressing TIMP2, an inhibitor of MMP9 [35]. Our findings in which MMP9 was increased 6.5-fold and TIMP-2 downregulated 15-fold in EOC are in agreement with these studies.

### Degradation of FBLN5

Our results indicate that *Fbln5* mRNA is downregulated in EOCs. Paradoxically, immunoreactive proteins to FBL5 antibodies were increased in EOC relative to benign ovarian tissues. We concluded that the immunoreactive proteins represented FBLN5 degradation proteins because all products were of decreased molecular size and were absent in tissue extracts incubated without FBLN5 antibodies (negative control blots). The mechanisms of downregulated *Fbln5* mRNA are not known but may represent cellular responses to FBLN5 fragments or as part of an abnormal transcriptome response. Nevertheless, downregulation of gene expression, together with protein degradation, favors loss of FBLN5 in EOC tumors.

Degradation of FBLN5 has been described in other pathological conditions. For example, Vierkotten, *et al.*, reported that degradation of FBLN5 led to fragmentation of Bruch's membrane and increased risk of age-related macular degeneration [36]. Hirai, *et al.*, showed that increased proteolytic cleavage in its N-terminal linker region with age, and that the truncated version is unable to promote elastogenesis [11]. Djokic *et al.* described proteolytic cleavage of fibulin-5 in the N-terminal linker region by a number of MMPs including MMP-2, −7, and −12 *in vitro* [37]. Interestingly, among fibulin proteases, MMP-7 was particularly potent [37].

### FBLN5 and EOC cell adhesion

Given its role in cell adhesion and metastasis in other cancers, we sought to understand the role of FBLN5 in EOCs using cell adhesion assays and three ovarian cancer cell lines developed at our institution. FBLN5 significantly decreased adhesion of the three cell lines tested to the ECM proteins laminin and collagen I. It should be emphasized that recombinant FBLN5 Gln^24^ – Phe^448^ was used for these assays which may not be folded physiologically due to lack of glycosylation. Nevertheless, this *in vitro* experiment suggests that FBLN5 may protect EOC metastasis by preventing cancer cell adhesion to specific ECM proteins.

The mechanisms by which FBLN5 prevented adhesion to collagen and laminin are not known. FBLN5 is known to bind to several cell types. For example fibulin-5 binds to integrins α9β1, αvβ3, and αvβ5 in CHO cells [38] and to α5β1 and α4β1 in aortic smooth muscle cells [16]. In general, β1, β3 and β5 subunits are highly expressed in ovarian cancer cell lines [39]) with β6 subunit present at varying levels. αvβ6 integrin is also highly expressed in EOC cell lines [40]. Since integrins α1β1, α2β1, α10β1, and α11β1 represent the predominant integrin receptors for collagen, fibulin-5 may not bind collagen directly. Potential interactions of fibulin-5 with collagen receptors discoid domain receptor (DDR) 1 and 2 have not been investigated although DDR1 is highly expressed in EOC [41, 42]. As for laminin, it is possible that fibulin-5 may compete with laminin receptors (αvβ3, αvβ5, α9β1). The finding that fibulin-5 inhibited adhesion of EOC cells to both laminin and collagen I might suggest the presence of other glycoproteins that have been suggested to enhance interactions between cell-matrix molecules by cross-linking a number of molecules including integrins, laminin, and collagens.

### Candidate proteases

We and others [11] have sought to understand proteolytic degradation of FBLN5. Previously, we identified an unknown serine protease increased in tissues from subjects with pelvic organ prolapse and a candidate serine protease that degraded FBLN5 *in vitro* [43]. Likewise, degradation of endogenous FBLN5 was shown to be increased in connective tissues of aging mice and mediated by an unknown serine protease [11]. Using *in vitro* assays and a candidate protease approach, we discovered two potential candidates that may degrade FBLN5 in EOC. First, a serine protease (purified porcine pancreatic elastase) degraded recombinant human FBLN5 within one minute to a 43 kDa product, similar size as the degraded fragment in EOC tumors. MMP7, however, was more potent and generated a degradation band of the expected size. By mass spectrometry, the cleavage site was identified in the linker region. Interestingly, a cleavage site similar to this was noted during purification of fibulin-5 [36] suggesting this site may be particularly vulnerable to proteolysis. Sequence specificity studies of several MMPs indicate that the P1’ site plays a critical role with little protease activity among peptides with a charged group or proline at P1’. Alanine at P1’ is most favored by MMP-1, −3, −7, and MMP-8 and compatible with FBLN5 A^94^ identified in the current study for MMP-7.

It is well-appreciated that tumor macrophages and MMPs contribute to progression of a number of malignancies [44], and much of their activities relate to remodeling of the ECM [45]. MMP7 has been shown to be overexpressed in human EOC [29, 46, 47]. MMP7 degrades many ECM proteins including collagens, proteoglycans, laminin, fibronectin, elastin and casein [28]. Additionally, MMP7 is able to convert proMMP2 to active MMP2 and proMMP9 to active MMP9 [29] thereby facilitating macrophage-tumor-stromal cell cross-talk. Overexpression of MMP7 has been shown to increase the invasiveness of several malignancies, including those of the colon and prostate [30]. Incubation of macrophages with malignant breast cancer cell lines, but not benign mammary epithelial cells, results in upregulation of macrophage MMP-7 [48]. We suggest that ovarian cancer cells stimulate MMP-7 production from tumor macrophages that readily degrade fibulin-5 thereby facilitating tumor progression and invasion. This idea is supported by the findings that reduced expression of FBLN5 correlates with poor prognostic features in hepatocellular carcinoma [21], and FBLN5 negatively regulates MMP-7 in these cells. FBLN5 may thereby exert its anti-metastatic function, in part, by down-regulating expression of MMP-7. Yue, *et al.* showed that FBLN5 suppressed lung cancer invasion by inhibition of MMP7 expression [5]. FBLN5 was silenced by promoter hypermethylation, and this resulted in activation of MMP7 and increased tumor invasion. Also, Wang, *et al.*, suggested that MMP7, which was stimulated in EOC by VEGF and IL-8, activated proMMP2 and proMMP9 which was associated with increased invasion of EOC cells [29].

In summary, we found dramatic upregulation of *MMP7* and FBLN5 degradative products in tumor macrophages of EOC. FBLN5 was shown to inhibit adhesion of EOC to matrix components. Together, the data provide insights regarding macrophage-induced regulation of the matrix microenvironment of EOCs that likely lead to increased adhesiveness of EOC metastatic spread.

## MATERIALS AND METHODS

### Human ovarian tissues

Benign human ovarian tissues were obtained from pre-menopausal women undergoing oophorectomy for reasons other than malignancy *n* = 12. Malignant ovarian tissue was obtained from patients undergoing cytoreductive surgery for metastatic epithelial ovarian cancer (*n* = 11). Biopsies were obtained primarily from metastatic sites. Tissues were snap frozen in liquid nitrogen, and stored at −80° C until use. The protocol for obtaining these tissues was approved by the Institutional Review Board of the University of Texas Southwestern Medical Center.

### RNA extraction

RNA was extracted as previously described [49]. Briefly, tissues were pulverized in liquid nitrogen and homogenized in RNA Stat-60 (TelTest Inc., Friendswood, TX, USA). Total RNA was then extracted according to the manufacturer's protocol, using chloroform (C2432, Sigma, St. Louis, MO, USA), isopropanol (I9516, Sigma, St. Louis, MO, USA), and ethyl alcohol (E190, Pharmaco-Aaper, Brookfield, CT, USA).

### cDNA synthesis

Reverse transcription was performed as previously described [50]. Reactions were conducted with 2 μg total RNA in a reaction volume of 20 μl. Each reaction contained 10 mM dithiothreitol, 0.5 mM deoxynucleotide triphosphates, 0.015 μg/ml random primers, 40 U RNase inhibitor (10777-019; Invitrogen, Carlsbad, CA, USA), and 200 U reverse transcriptase (18064-014; Invitrogen). Reaction conditions were 10 min at 23° C, 60 min at 50° C, and 15 min at 70° C.

### Quantitative real-time PCR

PCR reactions were performed in the manner previously described [51]. Reactions were carried out on an ABI Prism 7000 sequence-detection system (Applied Biosystems). The reverse transcription product from 50 ng RNA was used as the template, and reaction volumes (30 μl) contained Master Mix (Applied Biosystems). Primer concentrations were 900 nM. Cycling conditions were 2 min at 50° C, followed by 10 min at 95° C, then 40 cycles of 15 sec at 95° C and 1 min at 60° C. SYBR Green was used for amplicon detection. Gene expression was normalized to expression of the housekeeping gene, GAPDH, which was invariant among samples. A preprogrammed dissociation protocol was used after amplification to ensure that all the samples exhibited a single amplicon. Levels of mRNA were determined using the ddCt method (Applied Biosystems) and expressed relative to an external calibrator present on each plate. Primers used in this study are listed in Table 2.

**Table 2:** Primers used in this study

Gene	Accession #	Forward primer	Reverse primer	Probe
h*Elastin*	BC 065566	TGCAGCCTATAAAGCTGCTAAGG (330–352)	GGCACTTTCCCAGGCTTCA (455–437)	-
h*LOX*	AF 039291	GCGGCGGAGGAAAACTGT (964–981)	AGCAGCACCCTGTGATCATAATC (1037–1015)	-
h*LOXL1*	BC 068542	GACTGCCAGTGGATCGACATAA (1875–1896)	CTCCAAAACAATATACTTTGGGTTCA (1961–1936)	-
h*MMP2*	BC 002576	TTGATGGCATCGCTCAGATC (1690–1709)	TGTCACGTGGCGTCACAGT (1770–1752)	-
h*MMP9*	BC 006093	GAACTTTGACAGCGACAAGAAGTG (1136–1159)	GCCGCCACGAGGAACA (1204–1189)	-
h*COL1A1*	NM 000088	ACGAAGACATCCCACCAATCA (221–241)	CGTTGTCGCAGACGCAGAT (322–304)	-
h*COL3A1*	NM 000090	TCTTGGTCAGTCCTATGCGGATA (219–241)	CGGATCCTGAGTCACAGACACA (296–274)	6-FAM-AGATGT CTGGAAGCCAGA ACCATGCC-6-TA MRA (243–268)
h*TIMP1*	BC 000866	GACGGCCTTCTGCAATTCC (241–259)	GTATAAGGTGGTCTGGTTGACTTCTG (319–294)	-
h*TIMP2*	BC052605	CCCTCCTCGGCAGTGTGT (556–573)	CGGCCTTTCCTGCAATGA (628–611)	-
h*GAPDH*	BT 006893	GGAGTCAACGGATTTGGTCGTA (19–40)	CAACAATATCCACTTTACCAGAGTTA (94–69)	
		**ABI ref#**		-
h*Fib1*	NM_001996	Hs00972609		
h*Fib2*	NM_001004019	Hs00157482		
h*Fib3*	NM_001039348	Hs00244575		
h*Fib4*	NM_016938	Hs00973815		
h*Fib5*	NM_006329	Hs00197064		
h*Fib7*	NM_001128165	Hs00402230		
h*BMP1*	NM_001199	Hs00241807		
h*MMP7*	NM_002423	Hs01042796		

### Immunoblot analysis

Frozen ovarian tissues were pulverized with a liquid nitrogen-chilled mortar and pestle. Tissue powder was then homogenized in potassium phosphate buffer (16 mM potassium phosphate, pH 7.8, 0.12 M NaCl, 1 mM ethylenediaminetetraacetic acid) containing a protease inhibitor cocktail (Complete Mini, product number 11836153001, Roche Diagnostics, Penzberg, Germany), and then centrifuged at 10,000 *g* for 10 min. The supernatant (S1) was removed, and the previous homogenization step was repeated after resuspending the remaining tissue pellet in potassium phosphate buffer. After removal of the second supernatant (S2), the remaining tissue pellet was suspended in urea buffer (6.0 M urea in potassium phosphate buffer), homogenized, and extracted overnight at 4° C. Thereafter, the samples were centrifuged (13,000 *g* for 30 min), and the supernatant (S3) was removed. Protein concentrations were determined using a bicinchoninic acid protein assay (Pierce, Rockford, IL, USA) and standard curves of BSA in appropriate buffers. Total protein (30 μg/lane) was applied to 4 to 20% Criterion gradient polyacrylamide gels (Bio-Rad, Hercules, CA, USA), separated by electrophoresis, and transferred to nitrocellulose membranes overnight at 4° C. To ensure equal protein loading, identical gels were run side-by-side for Coomassie brilliant blue staining. Nitrocellulose membranes were placed in blocking buffer (10 mM Tris-HCl, pH 7.5, 0.15 M NaCl, 0.1% Tween 20, 2% nonfat powdered milk) for 1 h at 37° C and incubated with primary antibody (1:1000 dilution) for 1 h at 37° C. Primary antibodies were human fibulin 5 antibody (monoclonal mouse IgG1 Clone # 293904 derived from Gln^24^-Phe^448^ of recombinant human Fibulin 5 (epitope unknown, R&D Systems, Minneapolis, MN, USA) and a polyclonal antibody raised against full length rat FBLN5 that recognizes the human protein [23] (a kind gift from Dr. Elaine Davis, McGill University). Membranes were then washed with Tris-buffered saline solution (TBST) (10 mM Tris-HCl, pH 7.5, 0.15 M NaCl, and 0.1% Tween 20) for 3 min × 5. Thereafter, the blot was incubated with goat-anti-rabbit horseradish peroxidase conjugate (Bio-Rad, 1:10000 dilution) at room temperature for 1 h. The membrane wash protocol was repeated, followed by incubation with Western Lighting Chemiluminescence Reagent Plus (Perkin-Elmer, Boston, MA, USA) for 2 min. Chemiluminescence images were obtained on a Fuji LAS 3000 image analysis system (Fujifilm Life Science, Stamford, CT, USA).

### Gelatin zymography

Frozen ovarian tissues were pulverized with a liquid nitrogen-chilled mortar and pestle. Tissues were then homogenized in Tris buffer (10 mM Tris-HCl, pH 7.4, 150 mM NaCl, 10 mM CaCl_2_, 0.1% Triton X-100). Homogenates were centrifuged at 10,000 *g* for 15 min at 4° C. The supernatant was used for determination of protease activity. Protein concentrations were determined using bicinchoninic acid protein assay and standard curves of BSA in appropriate buffers. Samples (5 μg per lane) were applied to gelatin polyacrylamide minigels (Invitrogen, Carlsbad, CA, USA) (10%) in standard SDS loading buffer containing 0.1% SDS with no -mercaptoethanol; the samples were not boiled before loading. Gels were run at room temperature at 125 V. After electrophoresis, gels were soaked in renaturing buffer (2.7% [v/v] Triton X-100 in distilled water) in a shaker for 30 min with one change after 30 min to remove SDS. Next, gels were soaked in developing buffer (50 mM Tris, 200 mM NaCl, 5 mM CaCl_2_, 0.02% Brij 35, pH 7.5) overnight at 37° C and then stained with Coomassie brilliant blue-R 250 in 40% methanol and 10% acetic acid followed by washing with distilled water for 1 min. Clear zones of lysis against a dark background indicated enzyme activity. Areas of lysis were quantified using the Fuji LAS 3000 image analysis system.

### Cell adhesion assay

Three distinct epithelial ovarian cancer cell lines were used. All were developed by and provided by one of the authors (A.G.). The cell lines were developed from metastatic tumor ascites obtained at the time of cytoreductive surgery for advanced stage epithelial ovarian cancer from patients treated at the University of Texas Southwestern Medical Center (Table 1). Cells were cultured in ACL4 media (RPMI 1640 with insulin [0.02 mg/ml], transferrin [0.01 mg/ml], sodium selenite [25 nM], hydrocortisone [50 nM], HEPES [10 mM], epidermal growth factor [1 ng/ml], ethanolamine [0.01 mM], O-phosphorylethanolamine [0.01 mM], triiodothyronine [0.1 nM], bovine serum albumin [2 mg/mL], sodium pyruvate [0.5 mM] and L-glutamine [2.05 mM]) in a 37 C humidified incubator with 5% CO_2_. For the adhesion assay, a 48-Well CytoSelect Cell Adhesion Assay was used (Cell Biolabs, Inc., Catalog #CBA-070, San Diego, CA, USA). This kit consists of a 48-well cell culture plate pre-coated with six extracellular matrix proteins (fibronectin, collagen I, collagen IV, laminin I, fibrinogen and BSA). Half of the wells were additionally coated with 500 ng of recombinant human fibulin 5 (R&D Systems #3095-FB, Minneapolis, MN, USA) at a concentration of 10 μg/mL in PBS, and half were coated with 500 ng of BSA at a concentration of 10 μg/mL in PBS. These plates were allowed to air-dry under a sterile hood. Thereafter cells were plated according to the manufacturer's protocol using a cell suspension concentration of 1 × 10^6^ cells/mL. Cells were allowed to incubate for 2 hours prior to continuing the protocol.

### Proteolytic cleavage assays

Recombinant human fibulin 5 (R&D Systems #3095-FB, Minneapolis, MN, USA) was incubated with various proteases. All reactions were performed in a basic buffer (50 mM Tris-HCl, pH 7.4, 150 mM NaCl, 5 mM CaCl_2_, 0.1% TX-100, 0.01% brij L23). Enzymes were added to rhFib5 in the following molar ratios: 1:1.4, trypsin; 1:3.2, elastase, 1:8; thrombin, 1:17; MMP-9, 1:28; and 1:8 – 1:36, MMP-7. For the MMP9 assay, proMMP-9 (RayBiotech #228-11326) was incubated with 4-aminophenylmercuric acetate (APMA) at 37 C × 60 minutes to convert to active MMP-9. Thereafter, 10 ng of activated MMP-9 was added to 200 ng rhFib5 in a total of 20 μL of buffer for each time point (0, 2, 4, 6, 12 and 24 hours). Samples were boiled prior to loading on a 4 to 20% Criterion gradient polyacrylamide gels (Bio-Rad, Hercules, CA, USA). For thrombin, 0.01 U thrombin (Sigma T6884) was incubated with 200 ng rhFib5 in a total of 20 μL of buffer for each time point (0, 2, 4, 6, 12 and 24 hours). For MMP-2, 50 ng MMP-2 (PeproTech Cat. #420-02) was incubated with 200 ng rhFib5 in a total of 20 μL of buffer for each time point (0, 2, 2.5, 3, 3.5 and 4 hours). For trypsin, 50 ng trypsin (Sigma T4049) was incubated with 200 ng rhFib5 in a total of 20 μL of buffer for each time point (0, 2, 2.5, 3, 3.5 and 4 hours). Elastase assays were conducted with 25 ng of porcine pancreatic elastase (Sigma 45124) incubated with 200 ng rhFib5 in a total of 20 μL of buffer for each time point (0, 1, 12, 24, 36, 48 and 60 minutes). For the MMP-7 assay, 50 or 2.5 ng MMP-7 was incubated with 200 ng rhFib5 in a total of 20 μL of buffer for each time point (0, 10, 20, 30, 40, 50 and 60 seconds). Samples were boiled, loaded onto 4 to 20% polyacrylamide gels, separated by electrophoresis, transferred to PVDF membrane and immunoblotted as previously described.

### Mass spectrometry identification of degraded FBLN5

Protein gel pieces were digested overnight with elastase (Promega) following reduction and alkylation with DTT and iodoacetamide (Sigma-Aldrich). Then, samples underwent solid-phase extraction cleanup with Oasis HLB plates (Waters) and the resulting samples were analyzed by LC/MS/MS, using an Orbitrap Fusion Lumos mass spectrometer (Thermo Electron) coupled to an Ultimate 3000 RSLC-Nano liquid chromatography systems (Dionex). Samples were injected onto a 75 μm i.d., 50-cm long EasySpray column (Thermo), and eluted with a gradient from 1–28% buffer B over 40 min. Buffer A contained 2% (v/v) acetonitrile (CAN) and 0.1% formic acid in water, and buffer B contained 80% (v/v) ACN, 10% (v/v) trifluoroethanol, and 0.1% formic acid in water. The mass spectrometer operated in positive ion mode with a source voltage of 2.4 kV and an ion transfer tube temperature of 275° C. MS scans were acquired at 120,000 resolution in the Orbitrap and up to 10 MS/MS spectra were obtained in the ion trap for each full spectrum acquired using higher-energy collisional dissociation (HCD) for ions with charges 2–7. Dynamic exclusion was set for 25 s after an ion was selected for fragmentation.

Raw MS data files were converted to a peak list format and analyzed using the central proteomics facilities pipeline (CPFP), version 2.0.3 [52, 53]. Peptide identification was performed using a non-specific search with the open MS search algorithm (OMSSA) [54] against the appropriate protein database from Uniprot, with common contaminants and reversed decoy sequences appended [55]. Fragment and precursor tolerances of 20 ppm and 0.6 Da were specified. Carbamidomethylation of Cys was set as a fixed modification and oxidation of Met was set as a variable modification. At least two peptide spectral matches were required for a peptide to be considered confidently identified.

### Immunohistochemistry

Formalin-fixed paraffin-embedded tissues were sectioned at 4 microns and mounted on adhesive slides, along with multi-tumor sandwich block sections containing over 50 different normal and tumor tissues for external positive and negative controls [56]. After drying × 1 min in an 1100W microwave oven, slides were transferred to a 65° C oven and dried for an additional 20 min. Thereafter, slides were deparaffinized in xylene and rehydrated in graded alcohols to distilled water. Endogenous peroxidase activity was quenched for 10 min at room temperature using 0.3% H_2_O_2_ with 0.1% sodium azide. Primary antibodies were polyclonal fibulin 5 antibody [23] (1:500) in which antigen retrieval was conducted in 1mM EDTA, pH 8.5 × 30 min in a household vegetable steamer, followed by 10 min cool-down time. For CD68, slides were placed in 0.25 Tris base buffer, pH 9.0, in a pressure cooker. After rinsing in phosphate buffered saline (PBS) buffer, primary antibody incubation was performed for 50 min in a 25° C incubation oven, using gentle orbital rotation. Following another rinse in PBS, incubation with anti-rabbit or anti-mouse horseradish peroxidase-conjugated polymer (PowerVision Poly-HRP anti-rabbit IgG, Leica Biosystems, Buffalo Grove, IL, USA) was performed for 45 minutes at 25° C. Finally, slides were immersed for 8 minutes in 25° C diaminobenzidine (DAB) (Invitrogen, Carlsbad, CA), enhanced with 0.5% copper sulfate in PBS × 3 min, counterstained in hematoxylin, dehydrated in graded alcohols, cleared in xylene, and coverslipped. Negative controls were comprised of all treatments but without primary antibody.

### Statistical Analysis

Sample size for ovarian EOC immunoblotting was determined using pilot data of 12 benign and 4 ovarian tumors indicating that 10 tissues in each group were required for statistical significance at *P <* 0.05 with 80% power. Data are presented as mean ± SD for data derived from a single experiment and as mean ± SEM for data combined from two or more experiments. Student's *t* test with Bonferroni correction for multiple comparisons was used as appropriate or Fisher's exact as noted.
